# The OnyCOE-t™ questionnaire: responsiveness and clinical meaningfulness of a patient-reported outcomes questionnaire for toenail onychomycosis

**DOI:** 10.1186/1477-7525-4-50

**Published:** 2006-08-15

**Authors:** Lori P Potter, Susan D Mathias, Monika Raut, Farid Kianifard, Amir Tavakkol

**Affiliations:** 1 Ovation Research Group, San Francisco, CA, USA; 2 Ortho Biotech Clinical Affairs LLC, Bridgewater, NJ, USA; 3 Novartis Pharmaceuticals Corporation, East Hanover, NJ, USA

## Abstract

**Background:**

This research was conducted to confirm the validity and reliability and to assess the responsiveness and clinical meaningfulness of the OnyCOE-t^™^, a questionnaire specifically designed to measure patient-reported outcomes (PRO) associated with toenail onychomycosis.

**Methods:**

504 patients with toenail onychomycosis randomized to receive 12 weeks of terbinafine 250 mg/day with or without target toenail debridement in the IRON-CLAD^® ^trial completed the OnyCOE-t™ at baseline, weeks 6, 12, 24, and 48. The OnyCOE-t™ is composed of 6 multi-item scales and 1 single-item scale. These include a 7-item Toenail Symptom assessment, which comprises both Symptom Frequency and Symptom Bothersomeness scales; an 8-item Appearance Problems scale; a 7-item Physical Activities Problems scale; a 1-item Overall Problem scale; a 7-item Stigma scale; and a 3-item Treatment Satisfaction scale. In total, 33 toenail onychomycosis-specific items are included in the OnyCOE-t™. Clinical data, in particular the percent clearing of mycotic involvement in the target toenail, and OnyCOE-t™ responses were used to evaluate the questionnaire's reliability, validity, responsiveness, and the minimally clinical important difference (MCID).

**Results:**

The OnyCOE-t™ was shown to be reliable and valid. Construct validity and known groups validity were acceptable. Internal consistency reliability of multi-item scales was demonstrated by Cronbach's alpha > .84. Responsiveness was good, with the Treatment Satisfaction, Symptom Frequency, Overall Problem, and Appearance Problem scales demonstrating the most responsiveness (Guyatt's statistic of 1.72, 1.31, 1.13, and 1.11, respectively). MCID was evaluated for three different clinical measures, and indicated that approximately an 8.5-point change (on a 0 to 100 scale) was clinically meaningful based on a 25% improvement in target nail clearing.

**Conclusion:**

The OnyCOE-t™ questionnaire is a unique, toenail-specific PRO questionnaire that can be used with confidence in future studies of toenail onychomycosis. MCID was evaluated for three different clinical measures, and indicated that approximately a 7-point change (on a 0 to 100 scale) was clinically meaningful based on a 12.5% improvement in target nail clearing.

## Background

Onychomycosis is a common, chronic fungal infection of the keratinized tissue of the nail bed and nail plate that may impact patients' quality of life. Patients often experience pain and discomfort, and normal tactile functions can be impaired or lost. Toenail dystrophy can interfere with walking, standing, exercise, or proper shoe fit. Like other visible dermatologic imperfections, onychomycosis has both psychosocially and physically detrimental effects [1]. Patients report concerns about nail appearance, embarrassment, reduced self-esteem, and social withdrawal. Furthermore, patients may fear injury and spreading the infection to others [2].

To document the effect of onychomycosis on quality of life, Lubeck et al. [3] developed a patient-reported outcomes (PRO) questionnaire consisting of general and disease-specific measures (including symptom frequency and severity), which was used in a telephone study of 916 cases and 673 controls. Persons with toenail and/or fingernail onychomycosis reported statistically significantly lower quality of life scores on almost all measures, including pain, general health, social functioning, mental health, and functional limitation, compared with matched controls.

Subsequent studies have attempted to demonstrate the applicability of PRO results and their value to clinicians [4,5]. Following their initial research, Lubeck et al. conducted a validation study, in which they measured their questionnaire's responsiveness, or sensitivity to change, in a population of patients receiving usual and customary care for onychomycosis [4]. Changes in PRO scores were compared between patients who improved clinically and those who did not improve, based on the physician's subjective determination of clinical status at each visit. While onychomycosis-specific scales were responsive to clinical improvement, while no statistically significant changes were reported in a clinically stable group of patients [4].

Other studies of onychomycosis have had limited success linking PRO results to clinical status in onychomycosis. In a multinational study, Drake et al. [5] divided 532 toenail onychomycosis patients into two groups, based on the extent of nail involvement, and compared their quality of life scores. Patients with whole-nail involvement reported significantly lower quality of life scores than those with half-nail involvement. However, this study could not measure the responsiveness of PRO to changes in clinical status of patients.

In a telephone study of clinical trial participants, Drake et al. [6] attempted to correlate the quality of life scores with a patient-reported global severity rating. However, the study failed to show a clear relationship between quality of life factors (such as pain, discomfort, or psychosocial problems) and the overall severity rating, suggesting that severity and quality of life may be two distinct, non-overlapping measures.

The Drake studies illustrate some of the problems associated with clinical interpretation of PRO measures. Comparing PRO results to objective clinical assessment will provide a context for understanding the impairment in PRO associated with onychomycosis. Responsiveness and clinical meaningfulness are psychometric properties, which depend on clinical information, ideally objective information, such as a laboratory test result or quantifiable improvement. Moreover, there is a lack of prospective trial data in prior studies to demonstrate the impact of PRO on objective clinical measures associated with response to treatment of onychomycosis.

Thus, the objective of this current study was to fully validate the newly developed toenail-specific PRO questionnaire, the OnyCOE-t™, and in particular, to use objective clinical measures to assess its responsiveness and clinical meaningfulness, as part of a prospective trial of patients undergoing treatment for onychomycosis.

## Methods

### Study design and participants

Data were collected during the Improving Results in Onychomycosis – Concomitant Terbinafine (Lamisil^®^) and Debridement (IRON-CLAD^®^) trial, a prospective, open-label, randomized, multi-center study designed to evaluate the efficacy and safety of terbinafine. All eligible patients (N = 504) were randomized to one of two treatment groups; both groups received 12 weeks of terbinafine therapy, but only one group received aggressive debridement of the target toenail in addition to oral treatment. A randomization list was produced using a validated system that automates the random assignment of treatment groups to randomization numbers in a 1:1 ratio.

The study protocol was approved by a local institutional review board at each center and was carried out in accordance with Good Clinical Practice guidelines and the Declaration of Helsinki. Written informed consent was obtained from each subject prior to enrollment. Patients were aware of the reason for debridement, and the procedure was explained to them prior to obtaining consent. Debridement was performed at baseline, and weeks 6, 12, and 24 [Table 1].

**Table 1:** Patient Characteristics

	**Terbinafine Only**	**Terbinafine + Debridement**
	**N = 258**	**N = 246**
Male	64%	59%
White	63%	64%
Cured (Mycological)	60%	69%
Cure (Complete: Mycological + 100% Clearing)	38%	50%
Age ≥ 65	13%	17%
Mean Age	48.4 (± 12.9)]	49.9 (± 14.1)]

The IRON-CLAD^® ^study was designed to be inclusive (i.e., representative of onychomycosis patients who typically visit a podiatry practice). However, subjects with pre-existing liver disease, gastrointestinal malabsorption, nephropathy or blood disorders, liver or kidney dysfunction, and those with HIV or immunocompromized were excluded.

Study medications were provided to participants at no charge. Patient compensation was nominal and included the actual cost of transportation, parking, or other small expenses associated with participating in the study. No other compensation actual or implied was provided.

Patients self-administered the OnyCOE-t™ questionnaire at the time of enrollment (baseline) and at every scheduled visit for the duration of the study (Week 6, Week 12, Week 24, and Week 48), or upon early discontinuation from the study.

### Patient-reported outcome measures

The questionnaire was adapted from an existing validated instrument [3,4] that had been used to study onychomycosis of both the toenails and fingernails. It was revised to include only toenail-specific items; the new version of the questionnaire (the OnyCOE-t™ questionnaire) is composed of 33 items, with 6 multi-item scales and 1 single-item scale:

• A 7-item Toenail Symptom assessment, which comprises both Symptom Frequency (5 response categories: 1 = never, 5 = very often) and Symptom Bothersomeness scales (5 response categories: 1 = not at all bothered, 5 = extremely bothered)

• An 8-item Appearance Problems scale (4 response categories: 1 = very much a problem, 4 = not a problem)

• A 7-item Physical Activities Problems scale (4 response categories: 1 = very much a problem, 4 = not a problem)

• A 1-item Overall Problem scale (4 response categories; 1 = very much a problem, 4 = not a problem)

• A 7-item Stigma scale (5 response categories: 0 = does not describe me at all, 4 = describes me very well)

• A 3-item Treatment Satisfaction scale (5 response categories: 1 = very satisfied, 5 very dissatisfied)

For example, patients were asked: "During the past 4 weeks, how much of a problem were the following because of your nail fungal condition: Being embarrassed by the appearance of your nails?" or "Discomfort of pain from wearing shoes?" The response options ranged from 1 ("Very much a problem") to 4 ("Not a problem").

All items in the OnyCOE-t™ questionnaire were transformed to a 0 to 100 scale and scored so higher scores indicated better functioning. Each scale score was calculated as an average of all non-missing items if at least half of the items making up the scale were non-missing. Validation analyses were conducted to confirm that the revised questionnaire is valid and reliable and to assess responsiveness and calculate a minimally clinical important difference.

### Clinical outcomes

Clinical outcomes were based on mycology testing performed throughout the study (Culture and KOH results) and on examination of target toenail, specifically percent of the target nail judged clear of infection as well as the amount of new target nail growth observed.

Clinical assessments were performed by a well-trained, qualified person, other than the investigator; that person (nurse/study coordinator) remained blinded to debridement and used an assessment tool to avoid subjective evaluation. For the clinical assessment, the target nail was overlaid with a transparent film. The entire nail plate and the involved nail areas were outlined. The outline was transferred to a template fitted to the approximate size and shape of the nail. The template was divided into eight segments, each representing 12.5% of the nail. At each assessment, the number of segments involved (i.e., infected) was recorded. A segment was counted as infected only if ≥ 50% of the area of the segment was affected. The amount of clearing was determined by multiplying the number of infected segments by 12.5% and subtracting that percentage from 100%. For each patient, this process was performed by the same person at every visit.

Cure was designated in three ways. "Complete Cure" was defined as a negative culture and negative KOH, plus 100% clearing of the target nail. "Clinical Cure" was defined as ≥ 87.5% clearing of the target nail. "Mycological Cure" was defined as negative culture plus negative KOH. Cure status was determined at each clinic visit.

Improvement ("Improved") was defined as a positive change in the amount of clearing from baseline to end of study. "Not Improved" was defined as no positive change. In addition, a three-value categorical outcome of Improvement ("Improved", "Stable", or "Worsened") was used to compute the responsiveness of the questionnaire. Patients in the Stable group showed no change in the amount of clearing, and patients who Worsened showed negative change, or decrease in the amount of clearing.

Clinical assessment also included measuring new, clear nail growth by placing a cut at the proximal nail fold and measuring nail growth from that point onward. "Clinical Success" was determined by = 5 mm of clear new nail growth. Based on the study protocol, nail growth <5 mm at Week 48 was considered a therapeutic failure (i.e., not a Clinical Success).

### Confirmation of validity and reliability

Construct validity is assessed by examining the relationship between scales or items. Validity is demonstrated when scales or items that are thought to measure the same construct have high correlation coefficients; conversely, items assumed to measure different, unrelated constructs should have low correlation coefficients.

Known-groups validity evaluates the ability of a questionnaire to discriminate between groups by examining scores from subjects known to be different. In this study, the known-groups validity was evaluated using demographic and clinical criteria.

Internal consistency reliability measures the extent to which items within each scale correlate with each other to form a multi-item scale.

### Responsiveness

Responsiveness gauges the ability of a PRO measure to respond appropriately when a patient's clinical state changes. The analysis of responsiveness included patients who completed the OnyCOE-t™ questionnaire at baseline and at the end of the study and who had measures of clinical efficacy (clinical assessment of target nail clearing and re-growth). Change scores were computed for each scale by subtracting baseline scores from scores at the end of study (Week 48). Responsiveness was demonstrated when subjects who showed clinical improvement had greater, positive PRO change scores.

### Clinical meaningfulness

Clinical meaningfulness was analyzed by estimating the minimally clinical important difference (MCID) under different assumptions of nail clearing and new nail growth. The MCID was calculated based on differences of 12.5% in the degree of toenail involvement, which corresponded to the measurement method dictated by the protocol to evaluate the percent clearing of mycotic involvement. The MCID was also calculated based on a 25% difference in the degree of toenail involvement; increments of 25% were used in analysis of trial data to categorize severity of mycotic involvement at baseline. In addition to nail clearing, the MCID estimation was performed based on the Clinical Success criteria, i.e., ≥ 5 mm of clear new nail growth of the target nail.

### Statistical methods

Variable clustering was performed to evaluate the underlying structure of the theoretical OnyCOE-t™ scales and confirmed the existence of six multi-item scales [7]. Construct validity was limited by having no other scales to compare with the OnyCOE-t™ to establish external (criterion) validity. To test convergent validity, we generated Pearson's correlation coefficients between all items and scales, using pooled data.

Known-groups validity was assessed using a Student's t-test to compare change in patients who were cured (under all three definitions) versus those who were not; known groups was assessed using demographic characteristics as well.

Internal consistency reliability, a measure of the extent to which items within each scale correlate with each other to form a multi-item scale, was examined by computing Cronbach's alpha coefficient. A Cronbach's alpha coefficient of .70 or greater indicates good internal consistency of a multi-item scale [8].

In analyses of responsiveness, change scores were tested against zero using the one-sample t-test. Three groups of patients resulted from the categorical variable for Improvement; group scores were used to calculate Guyatt's statistic, the ratio of the mean change score for each group divided by the standard deviation for the Stable group. A statistic of 1.00 or greater (or -1.00 or less when improvement was denoted by a negative change score) was considered indicative of a measure highly responsive to change [9,10]. In addition, the difference in PRO scores between the Stable group and the Improved group was evaluated using the two-sample t-test.

For the estimation of the MCID, an analysis of variance (ANOVA) model for each OnyCOE-t™ questionnaire scale was fit using target toenail percent clearing or the amount of new growth of the target toenail as the single predictor variable. The least squares means estimate for each OnyCOE-t™ scale score was evaluated for every 12.5% or 25% difference in toenail clearing, and for clinical success as indicated by new toenail growth ≥ 5 mm.

For statistically significant comparisons (based on the p-values from the ANOVA model), the average difference in mean PRO scores between adjacent categories was used to estimate the MCID for each scale. The MCID calculation based on 12.5% difference included eight categories: 12.5%, 25%, 37.5% 50%, 62.5%, 75%, 87.5%, and 100%; the 25% difference included four categories: 25%, 50%, 75%, and 100%. However, when using the amount of new growth to estimate the MCID for each scale, we used the difference between the two categories (<5 mm, ≥ 5 mm).

All statistical analyses were generated using SAS Software, Version 8.2 or higher of the SAS System for Windows. No imputation (e.g., last-observation-carried-forward) was performed to accommodate missing assessments; analyses included only available data.

## Results

### Patient demographics

All patients were between 18 and 75 years of age, with a mean age of 48.5 years (terbinafine only) and 49.7 years (terbinafine plus debridement). The majority of patients were Caucasian (63.5%), and approximately two-thirds of patients were male (61.3%). Treatment groups were balanced with respect to patient characteristics [Table 1].

### Validity and reliability

Strong correlations exist between items measuring toenail symptom frequency, and bothersomeness. All the problem scales and items – Physical Activities, Physical Appearance, and Overall Problem – are moderately to highly correlated. Items expected to correlate highly, for instance those measuring constructs of embarrassment about appearance and social stigma, did. For instance, the Stigma scale and individual items correlated highly with both Problems with Activities and Problems with Appearance. Treatment Satisfaction correlates most highly with Symptom Frequency and Symptom Bothersomenesss.

We generally confirmed previous findings, demonstrating lower scores for females and for younger patients. Scale scores for cured versus uncured groups were significantly different, based on mycological cure. For instance, in the cured group (terbinafine plus debridement), the mean Symptom Frequency score was 84.2 vs 68.0 in the uncured group (p < 0.001); Overall Problems scores were 83.4 vs 56.4 (p < 0.001).

All scales demonstrated a high degree of internal consistency, as indicated by alpha coefficients > .70 (.84 to .91).

### Responsiveness

Patients in both groups (Improved and Not Improved) reported positive PRO change scores, as measured by change from baseline at Week 48 [[Table 2]. The Improved group demonstrated very good responsiveness, as shown by positive, statistically significant change in all measures. In the Not Improved group, Stigma and Treatment Satisfaction scores demonstrated no statistically significant change; all other PRO scores in the Not Improved group showed significant change, but gains were much smaller than those observed in the Improved group.

**Table 2:** Mean change in scores from baseline to week 48: Responsiveness based on clinical improvement (clearing of the target nail)

	**Clinically improved N = 400**	**Clinically not improved N = 52**
Symptom Frequency	34.5 (<.0001)	21.2 (<.0001)
Symptom Bothersomeness	24.1 (<.0001)	14.5 (.0022)
Physical Activities Problems	30.1 (<.0001)	11.3 (.0100)
Appearance Problems	34.4 (<.0001)	13.6 (.0013)
Overall Problem	44.0 (<.0001)	24.5 (<.0001)
Stigma	15.4 (<.0001)	5.1 (.1416)
Treatment Satisfaction	46.1 (<.0001)	1.7 (.8868)

(p-values are from the one-sample t-test)

Comparison of patients who improved to those who remained stable using Guyatt's statistic indicated that Treatment Satisfaction, Symptom Frequency, Overall Problem and Appearance Problem scales are all highly responsive, while all other scales were moderately responsive to clinical change [Table 3]. Moreover, results of two-sample t-tests showed statistically significant differences (p < .0001 to .0176) between the Improved group and the Stable group on all PRO scores except Symptom Bothersomeness (p = .3384) (data not shown).

**Table 3:** Guyatt's statistic and mean change in scales

**Scale**	**Worsened N = 12**	**Stable N = 37**	**Improved N = 400**
*Symptom Frequency*			
Mean Change	21.6	21.0	34.5
Guyatt's Statistic	.82	.80	1.31
*Symptom Bothersomeness*			
Mean Change	1.5	18.9	24.1
Guyatt's Statistic	.05	.64	.81
*Physical Activities Problems*			
Mean Change	18.3	9.0	30.1
Guyatt's Statistic	.55	.27	.91
*Appearance Problems*			
Mean Change	16.3	12.7	34.4
Guyatt's Statistic	.53	.41	1.11
*Overall Problem*			
Mean Change	22.2	25.2	44.0
Guyatt's Statistic	.57	.65	1.13
*Stigma*			
Mean Change	4.7	5.3	15.4
Guyatt's Statistic	.18	.20	.58
*Treatment Satisfaction*			
Mean Change	16.7	-2.1	46.1
Guyatt's Statistic	.62	-.08	1.72

### Clinical meaningfulness

Using the 12.5% definition, the MCID estimation for individual scales ranged from about 5.5 to 8 points, with the exception of the single item, Overall Problem, which had an MCID of about 11 points. Differences in the OnyCOE-t™ questionnaire scales at adjacent levels of improvement were not significant at all levels. The overall MCID using the 12.5% difference in clearing was 7.30 [Table 4].

**Table 4:** Minimal Clinically Important Difference

**Average Difference Between Adjacent PRO Scores**			
**Scale**	12.5% Difference in nail clearing*	25% Difference in nail clearing*	**≥ 5 mm new nail growth**^†^

Symptom Frequency	6.48	9.12	17.28
Symptom Bothersomeness	5.95	5.84	11.83
Physical Activities Problems	7.54	8.32	17.69
Appearance Problems	8.04	8.69	18.51
Overall Problem	11.19	11.77	25.32
Stigma	5.44	5.21	9.41
Treatment Satisfaction	7.42	9.11	16.37
**Overall**	**7.30**	**8.45**	**16.63**

* Based on comparisons that are significant at the .05 level

^†^Based on comparisons that are significant at the .0001 level

Under the 25% difference scenario, most of the individual MCID estimates were consistent (within a point) of the estimated MCID at 12.5%; the exceptions were Symptom Frequency and Treatment Satisfaction. Using 25% clearing to calculate MCID, all adjacent comparisons were significant. The overall MCID was 8.45 [Table 4].

The MCID assessment was calculated based on the clinical success criteria, i.e., ≥ 5 mm of clear new nail growth of the target nail [Table 4]. The Overall MCID for new nail growth was estimated as 16.63.

## Discussion

The goal of this study was to confirm the psychometric properties of the OnyCOE-t™ questionnaire as a tool for measuring PRO results specifically related to toenail onychomycosis, and to extend the prior research by evaluating responsiveness against an objective clinical measure and to calculate a measure of clinical meaningfulness [11]. Thus, this study focuses on presenting results of responsiveness and clinical meaningfulness, which interpreted the OnyCOE-t™ scores in clinically relevant terms. Results of validity and reliability analyses confirmed earlier research [4]. Additional analyses conducted showed the OnyCOE-t™ responded well to significant clinical changes in onychomycosis patients undergoing treatment.

It is important to note that clinical assessments were confined to the target nail, while PRO measures captured the patient's perception of the effects of disease and treatment on all nails affected. In a real-world scenario, this research artifact would not be present, and a clinician would be able to gauge a patient's overall clinical status as well as measure the patient's perception of his or her entire condition and treatment.

This validation study was conducted using data from a clinical trial in which both treatment groups received the same active medication, but only one treatment group received debridement in addition to medication. As expected, both groups improved and, clinically speaking, differences between the groups were small. As a result, the interpretation of results was challenging. Measuring responsiveness requires comparing a group expected to change with a group expected to remain stable [12]. However, based on our definition of improvement, nearly all patients (91%) improved by the end of the study. Even so, Treatment Satisfaction and Stigma change scores were significantly higher only among patients who demonstrated clinical improvement by the end of the study.

Even for other PRO measures, where change was significant in both the Improved and Not Improved groups, comparing the groups based on their respective amounts of change is instructive. For instance, the average change on the Physical Activities Problems scale in the Improved group was a 30-point gain, while the average change in the Not Improved group was only about a third as much, or 11 points.

Interpreting Guyatt's statistic was equally challenging because the Stable and Worsened groups were so small. Nevertheless, in the Improved group, most measures were responsive or highly responsive, while the Stable group showed no strong responsive measures.

Clinical meaningfulness is a way of translating a difference in a PRO measure into a difference in a clinical measure. In this study, target nail clearing and new nail growth provided a clinical basis for estimating the MCID for each scale, as well as for generating an overall MCID for the PRO questionnaire.

In the estimation of the MCID, the choice of a clinical measure is important, because a measure that captures too large a change in a patient's clinical status can obscure the PRO score's ability to reflect small but measurable changes in status. In this study, two measures of incremental improvement, based on target nail clearing, were evaluated.

Results for the smaller level of clinical change (12.5%) corresponded to the smallest average amount of change in the PRO scales (7.30); however, because the clinical differences were probably less discernible to patients (corresponding to one-eighth of a patient's target nail), not all of the adjacent comparisons were statistically significant and therefore not all differences were included in the estimation of the MCID. Estimation using 25% difference in nail clearing was based on statistically significant differences between all adjacent increments of clinical change. Nevertheless, results under both scenarios were consistent with respect to all scores except Symptom Frequency and Treatment Satisfaction.

In addition, a Clinical Success measure involving only two categories (Success or Failure, based on new nail growth of 5 mm or more) was used to estimate the MCID. Clinical Success encompassed a large amount of clinical improvement, and corresponded to a much larger MCID than percent clearing (The MCID for Clinical Success was 16.63, almost twice as large as the MCID for a 25% difference in nail clearing). The MCID is a way to quantify how sensitive a measure is to clinical change (i.e., how much does a scale need to change to correspond to a change in clinical status). For instance, based all three estimation methods, Stigma and Symptom Bothersomeness were the most sensitive measures: a 5- to 6-point gain in the scale corresponded to an incremental improvement in nail clearing (based on either 12.5% or 25% nail clearing improvement). Conversely, the Overall Problem measure reflected clinical change only after a gain of over 11 points, indicating a less sensitive scale.

In the absence of a clinical measure, the Minimally Important Difference (MID) can be estimated as some proportion of the standard deviation; a reasonable MID might represent one-quarter to one-third of a standard deviation for the score in question. In the context of baseline PRO scores, which had standard deviations of approximately 22 to 33 points, the overall MCID estimates for nail clearing represented amounts of change that generally fell into that range. The MCID for new nail growth fell outside that range – a MCID of 16.63 points represented one-half to three-quarters of a standard deviation of the baseline PRO scores.

The MCID can help interpret PRO results for the treating provider by translating an objectively understood clinical assessment, such as the percent of nail clearing, into a number of points on a PRO scale. For a clinician, this means that an average gain of about 8 to 9 points in PRO can be expected with a 25% improvement in nail clearing.

Future studies of the responsiveness and clinical meaningfulness of the OnyCOE-t™ questionnaire could address the limitations of the population. Administering this questionnaire to groups of patients expected to have different clinical outcomes (e.g., active versus placebo studies) might allow for sharper distinctions between Improved and Not Improved groups, and for a larger Stable group. Results on both measures of responsiveness would be more robust.

Both responsiveness and clinical meaningfulness provide clinicians with familiar frames of reference for understanding and interpreting PRO scores. In a broader context, the OnyCOE-t™ questionnaire will provide a tool, not only for researchers to assess PRO results from clinical trials, but also for managed care organizations to evaluate PRO measures of patients receiving treatment for onychomycosis. This study fills a gap in the literature by presenting the first validated instrument specific to toenail onychomycosis, and by demonstrating the relationship of the OnyCOE-t™ scales to clinical measures.

## Conclusion

The OnyCOE-t™ is a unique, toenail-specific PRO questionnaire that is responsive to clinical change and can be used with confidence in future studies of toenail onychomycosis.

## Competing interests

Financial support for this project was provided by Novartis Pharmaceuticals Corporation, East Hanover, NJ, USA. The IRON-CLAD^® ^trial was conducted, monitored, and data were collected by Novartis Pharmaceuticals Corp. The analysis for this study was conducted by Ovation Research Group.

Amir Tavakkol and Farid Kianifard are employees of Novartis Pharmaceuticals Corporation. Monika Raut is an employee of Johnson & Johnson, but was employed by Novartis Pharmaceuticals Corporation when this study was conducted. Lori P. Potter and Susan D. Mathias are employees of Ovation Research Group, a consulting firm, which received financial support for undertaking these analyses.

## Authors' contributions

LPP supervised the design of the validation study, performed the statistical analysis, and drafted the manuscript. SDM assisted in the design of the validation study, participated in the design of the statistical analysis, and helped to draft the manuscript. MR participated in the design and coordination of the validation study and helped to draft the manuscript. FK participated in the design of the statistical analysis and assisted in interpreting results. He also designed the statistical plan and analysis of IRON-CLAD^®^. AT designed the IRON-CLAD^® ^trial and supervised its analysis, provided advice on the design of the validation analysis, and helped to draft the manuscript. All authors read and approved the final manuscript.

## References

[B1] Elewski BE (1997). The effect of toenail onychomycosis on patient quality of life. Int J Dermatol.

[B2] Scher RK (1994). Onychomycosis is more than a cosmetic problem. Br J Dermatol.

[B3] Lubeck DP, Patrick DL, McNulty P, Fifer SK, Birnbaum J (1993). Quality of life of persons with onychomycosis. Qual Life Res.

[B4] Lubeck DP, Gause D, Schein JR, Prebil LE, Potter LP (1999). A health-related quality of life measure for use in patients with onychomycosis: a validation study. Qual Life Res.

[B5] Drake LA, Patrick DL, Fleckman P, Andr J, Baran R, Haneke E, Sapede C, Tosti A (1999). The impact of onychomycosis on quality of life: development of an international onychomycosis-specific questionnaire to measure patient quality of life. J Am Acad Dermatol.

[B6] Drake LA, Scher RK, Smith EB, Faich GA, Smith SL, Hong JJ, Stiller MJ (1998). Effect of onychomycosis on quality of life. J Am Acad Dermatol.

[B7] Pasta DJ, Potter LP (1996). Exploratory Variable Clustering for Integrating Analyses: San Francisco, CA..

[B8] Cronbach LJ (1951). Coefficient alpha and the internal structure of tests.

[B9] Guyatt GH, Bombardier C, Tugwell PX (1986). Measuring disease-specific quality of life in clinical trials. Cmaj.

[B10] Guyatt G, Walter S, Norman G (1987). Measuring change over time: assessing the usefulness of evaluative instruments. J Chronic Dis.

[B11] Potter LP, Mathias SD (2005). The OnyCOE-t Questionnaire: Reliability and Validity of a Patient Reported Outcomes Questionnaire: October 19-22; San Francisco, CA..

[B12] (2002). Assessing health status and quality-of-life instruments: attributes and review criteria. Qual Life Res.

